# Modular instrument actuation unit for robotic-assisted systems in laparoscopic surgery

**DOI:** 10.1007/s11548-026-03728-7

**Published:** 2026-06-20

**Authors:** Luca Fäth, Sonja Stabenow, Dennis N. Schneider, Alexander Kirst, Dirk Wilhelm

**Affiliations:** 1 School of Medicine and Health, Technical University of Munich, Munich, Germany; 2 School of Engineering and Design, Technical University of Munich, Munich, Germany; 3 German Aerospace Center, Institute of Robotics and Mechatronics, Weßling, Germany; 4 Department of Surgery, Technical University of Munich, School of Medicine and Health, TUM University Hospital, Munich, Germany

**Keywords:** Robotic-assisted surgery, Modular Toolhead, Laparoscopic instruments, Autonomy

## Abstract

****Purpose**:**

Minimally invasive surgery involves repetitive and physically demanding manipulation tasks that contribute to surgeon fatigue and limit ergonomic efficiency. Semi-autonomous robotic assistance offers the potential to alleviate this workload, particularly for non-destructive supporting actions, such as tissue traction and presentation. This work presents a modular robotic toolhead designed to actuate standard laparoscopic instruments on robotic platforms, providing a research platform for autonomous assistance.

****Methods**:**

The developed system integrates grasping actuation with a novel capstan linear drive, axial instrument rotation, extensible electrical interfaces, and a ROS2-based software framework into a compact end-effector without requiring instrument modification. The toolhead prototype undergoes functional testing and setup evaluation in a clinical simulation.

****Results**:**

Functional verification included successful grasping and axial rotation of different laparoscopic grasper types, insertion and removal of an instrument via a dedicated interface, exchange of the complete instrument adapter and toolhead, and decoupling under excessive grasping force. The software framework enabled coordinated actuation control, system monitoring, and data acquisition for future evaluation. The integration of the system into a laparoscopic phantom setup representative of cholecystectomy and sigmoid resection procedures showed workspace compatibility and workflow integration.

****Conclusions**:**

The results demonstrate the feasibility of the proposed modular architecture as a flexible research platform for robotic laparoscopic assistance and future investigation of force-controlled grasping and semi-autonomous manipulation strategies.

## Introduction

Minimally invasive surgery (MIS) requires surgeons to perform complex manipulation tasks using elongated instruments introduced through trocars placed in small incisions. While MIS offers well-established clinical benefits, including reduced patient trauma and faster recovery [1], it also imposes considerable physical and cognitive demands on the surgeon [2]. Despite ongoing advancements in surgical technique and technology, this workload remains a substantial contribution to fatigue and variability in performance, particularly during prolonged procedures [3]. Robotic surgical systems have demonstrated the potential to mitigate several of these challenges by improving surgical precision and ergonomics [4, 5]. Commercial platforms such as the daVinci system developed by Intuitive Surgical [6] enable motion scaling [7] and tremor reduction [8] and have shown clinical benefits across a variety of procedures. While improving surgical outcomes and supporting more ergonomic surgeon operation, adoption of these systems is typically associated with high acquisition and operating costs, reliance on proprietary hardware and software architectures [9], and limited modularity and adaptability [10]. As a result, their accessibility remains restricted, and integration into diverse clinical workflows and research settings is often challenging. With current commercial systems being predominantly limited to teleoperated control and not yet natively supporting autonomous or semi-autonomous functionalities [11], their potential for workflow automation and intelligent surgical assistance is highly restricted.

We bridge this gap by enabling the direct usage of standard laparoscopic instruments in a robotic setting. This is a crucial step, since widespread adoption and proven reliability of standard laparoscopic instruments make them an attractive basis for investigating robotic assistance and autonomous support concepts, particularly for economic and logistical considerations. Rather than redesigning the distal end, this approach enables autonomous control through a modular actuation unit. The mechanical properties of the instrument are then identical to those used in operating rooms, allowing research insights to transfer directly from current surgical practice to research on autonomous systems, decoupled from any specific proprietary robotic platform. From a functional perspective, tissue retraction and exposure represent intuitive entry points for robotic assistance, as these tasks are repetitive, physically demanding, and do not result in destructive operations, thus being repeatable in a research setting [12].

We focus on the development of a modular robotic toolhead that allows standard laparoscopic instruments to be actuated by a cost-efficient, adaptable platform, designed for safe operation in shared human–robot workspaces, without requiring modification of the instruments themselves. The system is exemplified through integration with a 7-degree-of-freedom robotic arm and is designed to preserve established surgical workflows while enabling robotic execution of secondary manipulation tasks. The surgeon retains full authority over the procedure at all times and can supervise, intervene, or override robotic motions as required.

**Fig. 1:**
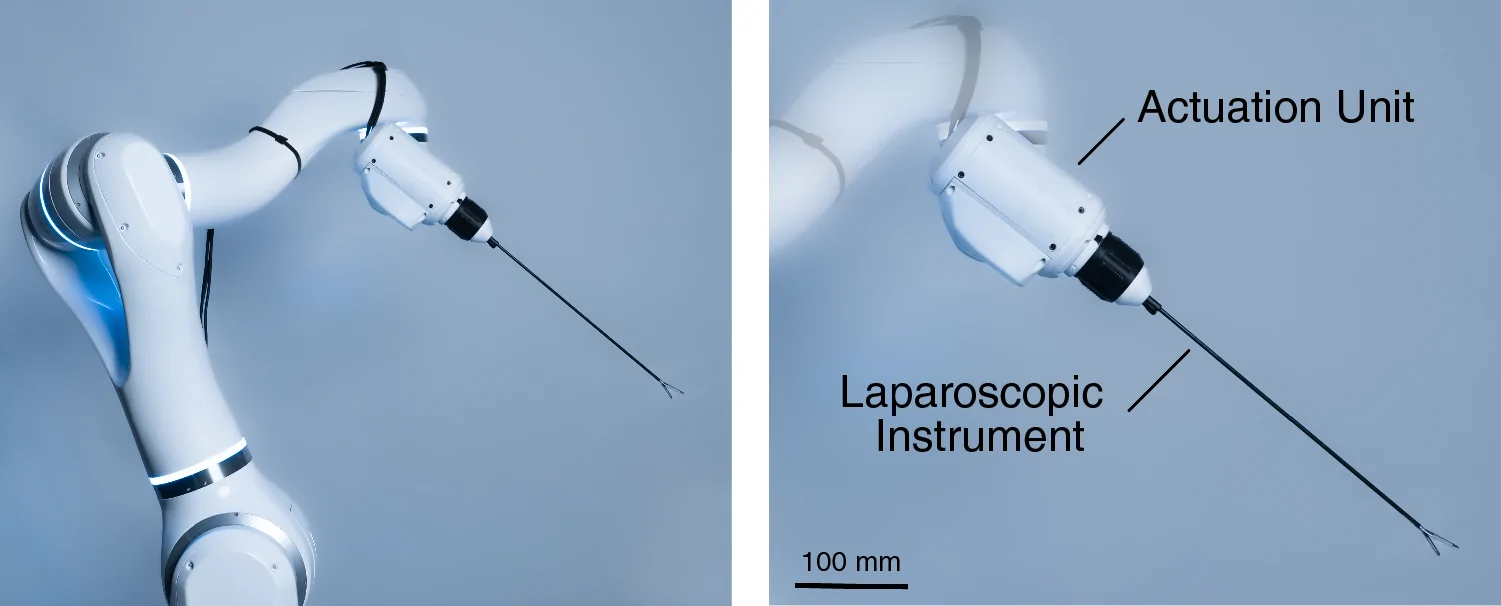
Robot base and mounted instrument actuation unit with the inserted standard laparoscopic tool

The focus of this work is on system design, integration, and feasibility. By providing a compact, modular end-effector platform for controlled grasping and instrument rotation, our work establishes a foundation for future quantitative evaluation and the investigation of higher-level autonomous assistance in minimally invasive surgery with the system shown in Fig. 1.

### Related work

Work with standard laparoscopic instruments is widespread [10, 13, 14]. Previous work has investigated robotic handling and force-aware actuation of standard laparoscopic instruments. Khadem et al. [15] present a force-controlled laparoscopic instrument that integrates actuation and proximal sensing to regulate pinch force under externally applied loads. Their work demonstrates reliable grasp-force control using standard instruments within a dedicated instrument-drive concept. In their work, the actuation of the rotation is integrated into the robot base, making the grasping modular but reducing transferability to other robotic systems.

A broader perspective on robotic instrument handling is provided by Schäfer and colleagues, as summarized in Schäfer’s dissertation [16], which consolidates multiple contributions on extracorporeal force estimation, haptic feedback, and robotic laparoscopic assistance [10, 17–19]. Early implementations employ standard laparoscopic instruments mounted on a dedicated toolhead attached to a robotic arm, while later developments introduce a more integrated proprietary platform with additional distal degrees of freedom. These contributions provide valuable reference points regarding feasible force ranges, the practicality of current-based proximal force estimation, and relevant system-level considerations. However, the inclusion of a passive gimbal joint can introduce position-dependent control characteristics, which potentially complicate the implementation of automated trajectories.

## System concept

The modular research platform interfaces MIS instrumentation with a robotic system via a mechatronic toolhead. The movement range is designed to be integrated into a shared robot-surgeon workspace without obstructing the surgeon’s movement. More specifically, the system design focuses on tissue retraction as a representative supporting task. It is structured to accommodate variations in surgical use cases, such as differences in patient positioning and trocar placement for laparoscopic cholecystectomy and sigmoid resection.

The concept is based on the objective of enabling autonomous robotic support through learning surgical skills from practice where standard laparoscopic instruments are extensively utilized. Using standard clinical instruments creates a procedurally relevant framework that allows research insights to be directly mapped to and from clinical practice. Therefore, the toolhead concept is built around the usage of standard laparoscopic tools while considering universal actuation principles that can be adopted for further work. The concept consequently includes the actuation of the single degree of freedom of the laparoscopic instrument.

The platform for positioning relative to the operating table is a robot arm mounted on a movable platform. The robot’s kinematic redundancy allows for flexible configuration around trocar constraints and facilitate safe operation in proximity to clinical personnel. Inline mounting of the instrument along the robot’s terminal joint axis would result in excessive overall length due to the distal joint geometry and the added extension of the actuation unit, rendering this configuration impractical for collaborative surgical use. In the observed surgical procedures, the instrument handle is often positioned close to the surgeon’s body during manipulation, reinforcing this consideration. The toolhead is therefore mounted at a $90^\circ$ offset relative to the robot flange. Consequently, the instrument’s roll axis is no longer aligned with the robot’s terminal joint axis, and axial rotation would otherwise require coordinated motion of the robotic arm. Since this is a frequent motion in surgical manipulation, a dedicated rotational axis is integrated into the toolhead to provide direct actuation while minimizing unnecessary whole-arm motion.

The work comprises a two degree of freedom actuation unit that integrates standard laparoscopic tools to form an end effector; this is interfaced with a robotic platform and a modular software framework to support research on robotic assistance and autonomy in minimally invasive surgery.

## Methods

### System overview

**Fig. 2:**
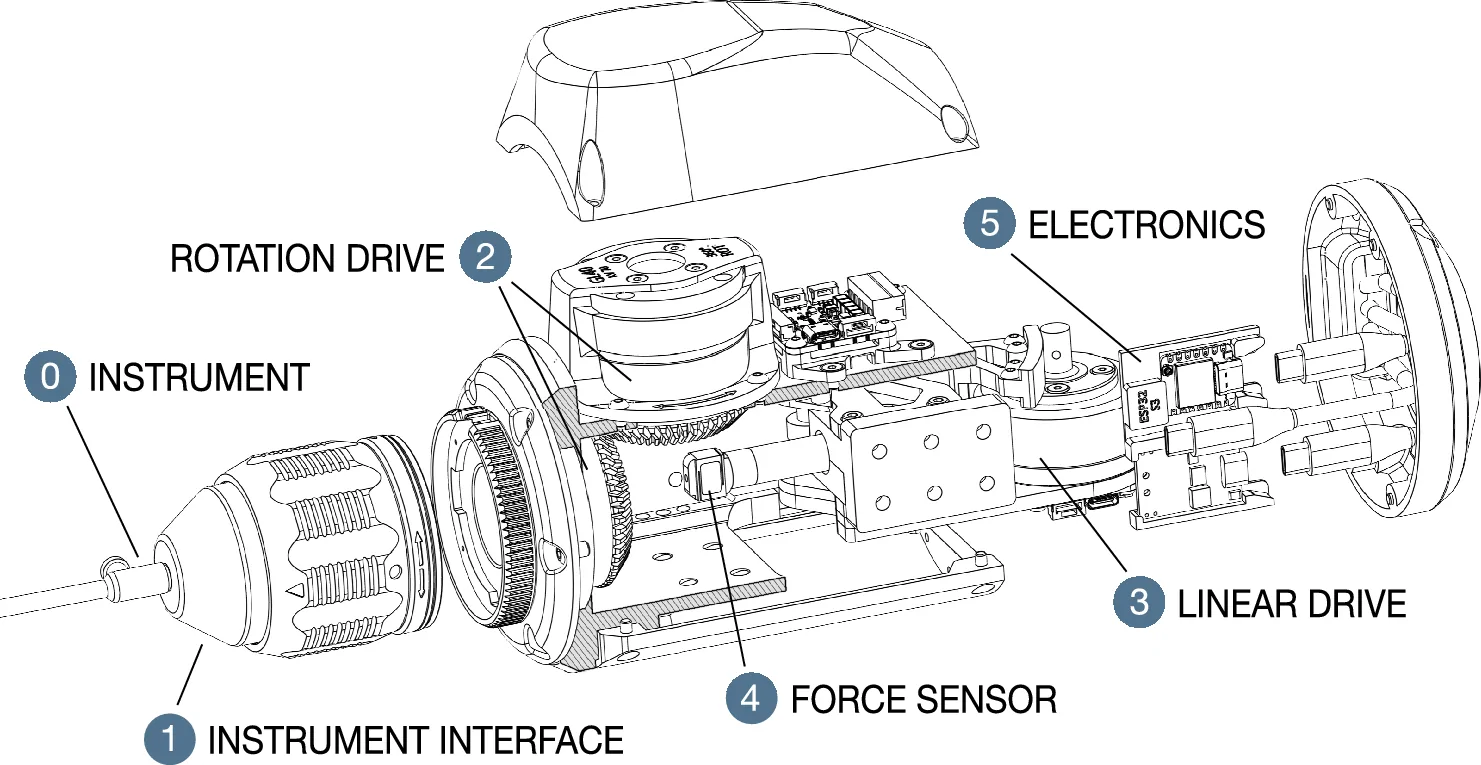
Toolhead subsystem architecture, showing the major functional modules integrated into the housing structure: instrument interface, linear and rotational drives, force sensing, and electronics

The system configuration consists of a standard robotic arm equipped with the modular robotic toolhead and a standard laparoscopic instrument mounted to it. In the implementation, a MAiRA S robotic arm from Neura Robotics is used as the carrier platform, while all task-specific actuation, sensing, and instrument interaction are implemented at the toolhead level. The toolhead is mounted to the robot flange via a custom mechanical adapter and forms the functional core of the system. Mechanical, electrical, and software interfaces between the robot and the toolhead are defined in a platform-agnostic manner, enabling independent development and adaptation of the toolhead [20]. Illustrated in Fig. 2, the toolhead is composed of multiple functional subsystems, including the instrument interface, grasping actuation, in-axis rotation, sensing components, and electronics.

These subsystems are integrated into a rigid structural housing that provides mechanical stability, reliable load transfer, and structural modularity, allowing individual subsystems to be adjusted or replaced without requiring fundamental changes to the remaining system. The laparoscopic instrument is inserted into the toolhead through a dedicated instrument interface. This interface mechanically couples the instrument to the actuation and sensing chain, enabling the transmission of linear push–pull forces to the instrument’s actuation pin for opening and closing the grasper, as well as for rotation about the instrument’s longitudinal axis. Interaction forces arising from contact with tissue, the patient, or the trocar are transmitted through the toolhead to the robot flange, where they can be measured by the robot-integrated force–torque sensor. Grasping actuation and in-axis instrument rotation are implemented locally within the toolhead and can operate independently of the robot’s joint-level motion. Force-related information is obtained from actuator-side motor current measurements. An inline force sensor is prepared for integration into the toolhead; full mechanical integration and closed-loop force control are addressed as future work. Electronic components integrated into the toolhead provide power distribution, sensing, communication, and low-level control. Power is supplied externally and is internally regulated, with separate supply domains for actuation and low-voltage electronics. High-level commands are provided by a host system running ROS 2, while low-level actuator control and sensor acquisition are handled locally by the motor controllers [21]. The software architecture follows a modular ROS 2-based framework, providing interfaces for system monitoring, user feedback, and surgeon intervention.

#### Instrument interface

**Fig. 3:**
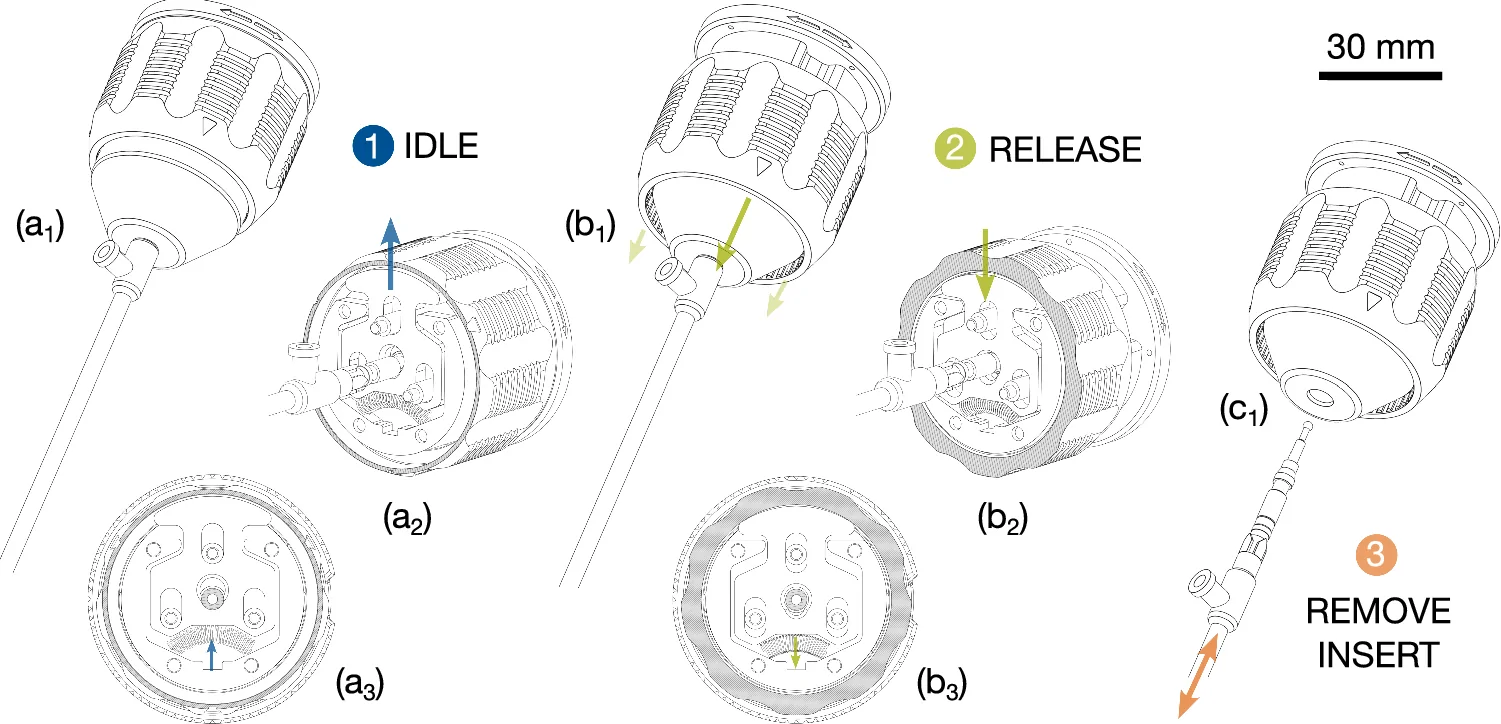
Insertion and release mechanism of the Instrument: **a** idle state, **b** instrument release with knob pulled, **c** instrument removal, illustrating the spring-loaded locking principle

The instrument interface provides the mechanical and functional coupling between the toolhead and standard laparoscopic instruments, ensuring repeatable alignment, secure locking, and the reliable transmission of loads and actuation forces. Instrument insertion and removal are realized via a spring-loaded locking mechanism integrated into the instrument interface adapter illustrated in Fig. 3. During insertion, the instrument is guided along its insertion axis until a spring-loaded sliding lock disk engages a circumferential feature of the instrument shaft, securing the instrument axially. Actuating the adapter sliding knob displaces the locking disk against the spring via an internal ramp, enabling instrument removal while the adapter remains attached. In addition to structural fixation, the instrument’s proximal actuation pin is coupled to a sliding actuation element within the interface. This coupling is designed to engage synchronously with the main instrument lock, ensuring that mechanical fixation and actuation coupling are established simultaneously. The interface transmits a single linear degree of freedom to actuate grasping of the inserted instrument.

A bayonet-style connector, requiring an axial engagement and lock, couples the complete instrument adapter assembly to the rotational drive. This enables two exchange modes: rapid instrument exchange via the spring-loaded locking mechanism, and removal of the entire instrument adapter assembly via the bayonet interface. The latter supports modular adaptation to alternative instrument geometries and provides a basis for automated tool exchange through dedicated robot maneuvers. The linear actuation of the instrument’s internal actuation rod is coupled to the instrument interface through a magnetic coupling. This coupling engages automatically when the instrument adapter assembly is mounted and transmits push–pull forces to the instrument actuation pin. Pulling forces correspond directly to closing of the grasper jaws and therefore determine the applied grasping force at the tissue. To introduce a passive safety mechanism, the magnetic coupling is designed to disengage when tensile forces exceed a defined threshold (approximately 60 N in the current prototype). As a result, the maximum grasping force is mechanically limited, complementing software-based force constraints. Pushing forces, which correspond to opening of the grasper, do not lead to disengagement. The pushing motion is instead bounded in range by the geometry of the interface and instrument, preventing over-opening and associated damage. After disengagement, the magnetic coupling automatically re-engages once the linear drive returns to a safe position, without requiring manual intervention. In the event of power loss, control failure, or other system interruptions, the instrument can be detached from the robotic toolhead and transferred directly to a standard manual handle, allowing continuation of the surgical procedure.

#### Instrument rotation

**Fig. 4:**
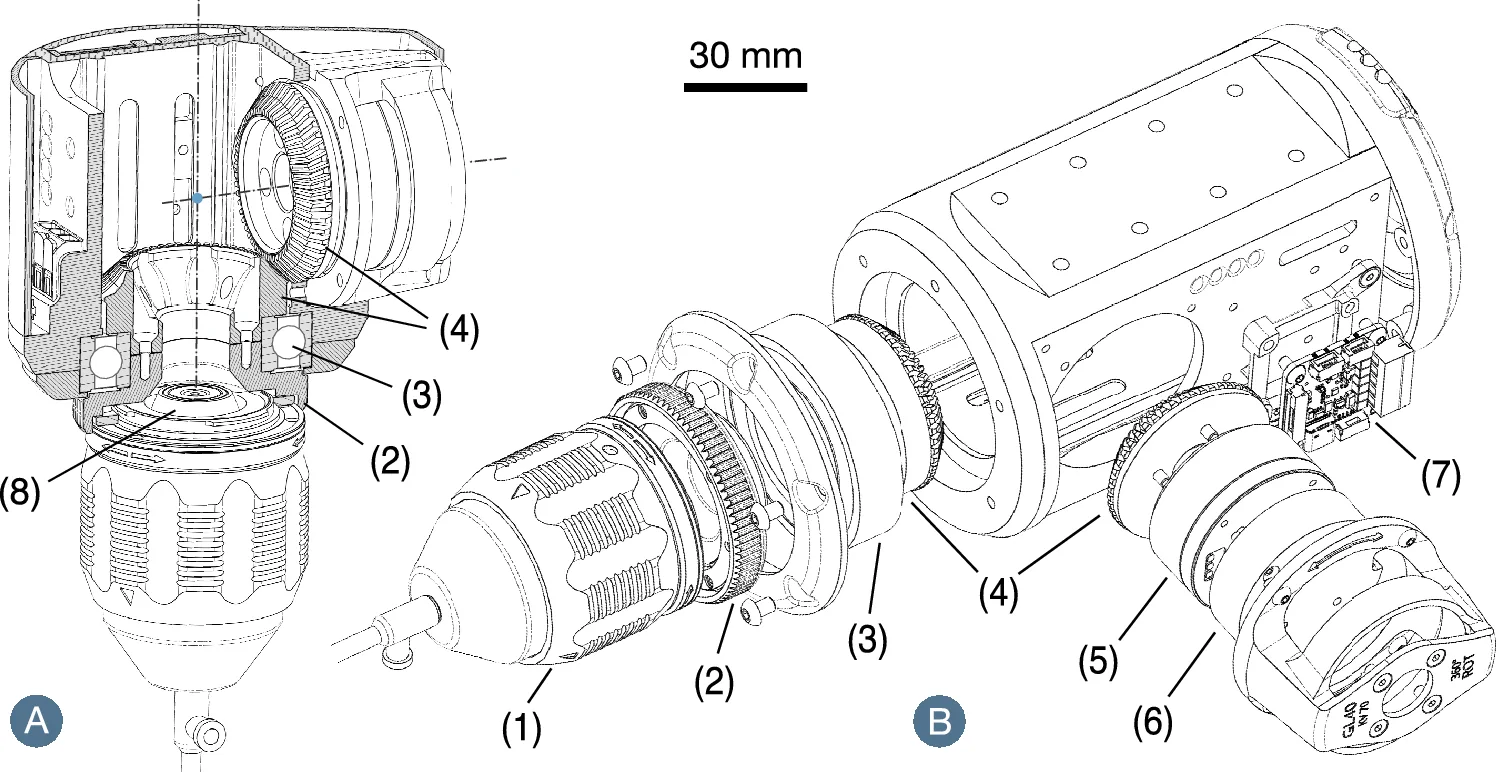
**A** Sectional and **B** exploded view of the rotation drive subsystem showing (1) instrument adapter, (2) bayonet connector, (3) bearing support, (4) bevel-gear transmission, (5) motor, (6) magnetic encoder integrated in the motor housing, (7) motor controller, and (8) magnetic coupling for the linear grasping actuation passing through the hollow shaft

The rotational drive provides controlled rotation of the laparoscopic instrument about its longitudinal axis. This additional degree of freedom enables precise axial orientation of the instrument during tissue manipulation. The corresponding subsystem, illustrated in Fig. 4, incorporates a central passage for the linear grasping actuation chain and associated sensing elements. To accommodate this layout with a compact overall form factor, axial rotation is realized using a $90^{\circ }$ bevel gear stage with a hollow output shaft, resulting in a coaxial configuration that enables rotational and linear actuation to be combined without mechanical interference.

Axial rotation is achieved by rotating the entire instrument adapter assembly, which is mechanically coupled to the rotational drive via the previously described bayonet interface. The rotating assembly is supported by a bearing arrangement integrated into the structural housing of the toolhead, ensuring stable alignment of the instrument axis and reliable transmission of torsional loads, while leaving the hollow shaft’s central passage clear for the linear actuation chain. The bevel gear stage implements a 1:1 transmission ratio between the drive motor and the rotating output. Torque is provided by a brushless DC motor integrated into the toolhead, with rotational position feedback obtained from a magnetic encoder in the motor housing sensing a shaft-mounted magnet. The encoder feedback is used as a proxy for the output rotation angle. While instrument axial rotation does not require motion of the robotic arm joints, it can be synchronized with higher-level robot motion through the ROS 2-based control framework. This allows coordinated execution of instrument rotation and global positioning when required, while still enabling frequent axial adjustments to be performed without inducing unnecessary robot arm motion or additional interaction forces at the trocar. The linear grasping actuation mechanism, which passes through the rotational stage, is described in the following section.

#### Grasping actuation

**Fig. 5:**
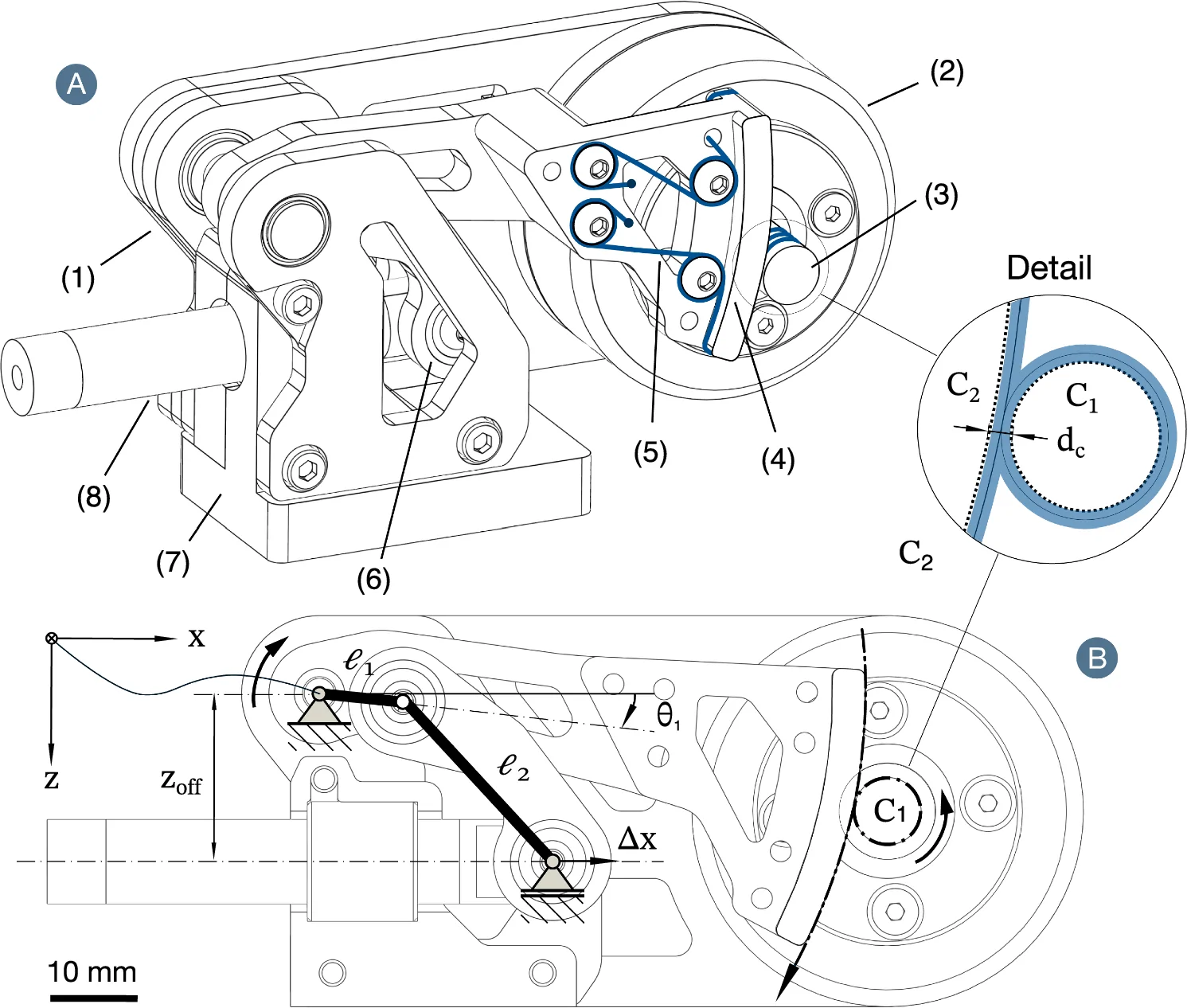
**A** Overview of the Capstan Linear Drive module showing (1) structural frame, (2) motor, (3) small capstan, (4) large capstan, (5) cable transmission, (6) connecting link, (7) mounting block with linear guide, and (8) push-pull rod for instrument grasping actuation. **B** Geometric model of the capstan–linkage mechanism with parameters used for analytical derivation of kinematics and force transmission

The grasping actuation subsystem illustrated in Fig. 5A provides controlled linear motion for opening and closing of the laparoscopic instrument jaws. Kinematic characterization of the selected instruments shows, a linear stroke of approximately $2\,\textrm{mm}$ is required to cover the full functional range of the two examined grasper variants. The linear drive converts rotary motor motion into precise, small-scale linear displacement, enabling controlled force application.

The actuation module is designed to be configurable to empirical requirements derived from iteratively refined force–stroke characteristics, enabling parameter changes at the transmission and linkage level. To support this flexibility, the toolhead structure provides adjustable mounting of the linear drive module, allowing the engagement position to the instrument actuation interface to be adapted for alternative concepts, inline force sensor integration, and transmission parameter adjustments.

The drive architecture combines a capstan-based reduction stage with a compact two-link rotary-to-linear conversion mechanism. A brushless DC motor drives a small input capstan, which is coupled to an output capstan via a tensioned braided Dyneema microfiber fishing line (0.4 mm diameter). For the current 3D-printed prototype, this commercially available low-stretch line was selected as a practical approximation of the high-modulus synthetic fiber ropes established for capstan drives in robotic systems [22, 23], based on its manufacturer-specified stretch-free characteristics, high tensile load capacity relative to its diameter, and compatibility with the compact capstan geometry. The 0.4 mm diameter was chosen to minimize the effective capstan radius offset introduced by rope compression while maintaining sufficient force transmission capacity. The resulting angular motion is converted into linear displacement through a linkage connected to a guided push rod. This push rod interfaces with the instrument actuation pin via the magnetic coupling described.

All geometric parameters appearing in Eqs. (1)–(7) are defined in Fig. 5B. The capstan stage provides an angular reduction that is nominally determined by the ratio of the capstan circumferences,

1 $$\begin{aligned} \rho _{\textrm{nom}} = \sigma \frac{C_1}{C_2}, \end{aligned}$$

where $C_1$ and $C_2$ denote the nominal circumferences of the input and output capstans and σ indicates the wrap direction. In the assembled system, deviations from the nominal ratio occur due to rope diameter, elastic deformation, and manufacturing tolerances. These effects are captured by an experimentally identified effective transmission ratio $\rho _{\textrm{eff}}$, used for the motor-to-linkage mapping:

2 $$\begin{aligned} \theta _1 = \rho _{\textrm{eff}}\,\varphi , \end{aligned}$$

in which φ is defined as the motor angle and $\theta _1$ as the angle of the linkage input.

The subsequent two-link mechanism transforms the linkage input angle into linear motion according to

3 $$\begin{aligned} x(\theta _1) = \ell _1 \cos \theta _1 + \sqrt{ \ell _2^2 - \bigl ( z_{\text {off}} - \ell _1 \sin \theta _1 \bigr )^2 }, \end{aligned}$$

with $\ell _1$ and $\ell _2$ representing the linkage lengths and $z_{\text {off}}$ the geometric offset of the linear guide. Together, Eqs. (2) and (3) define the motor-to-slider mapping x(φ) and allow the stroke, resolution, and mechanical advantage of the actuation to be shaped through geometric parameter selection. The linkage geometry was intentionally designed such that the mechanical advantage increases as the jaws close, counteracting the reduced force transmission coefficient of the laparoscopic instrument’s four-bar grasping mechanism at small jaw angles [14], thereby providing a more effective grasping force across the functional range.

For the implemented prototype geometry, the usable operating range corresponds to an effective linear stroke of up to $4\,\textrm{mm}$, achieved over approximately one full motor revolution, which exceeds the $2\,\textrm{mm}$ functional requirement of the examined instrument variants. This reserve stroke serves three purposes: it provides compliance compensation headroom; it accommodates instrument variants with larger stroke requirements than the examined graspers; and it allows the engagement position within the nonlinear transmission curve to be selected as an additional tuning parameter—shifting the operating window adjusts the effective mechanical advantage profile across the grasping range without requiring geometric changes to the linkage itself. Mechanical hard stops and software-defined limits restrict the operating range to maintain a safety margin from geometric singularities and overtravel conditions. The same kinematic mapping enables estimation of the linear output force based on actuator-side measurements. The motor controller provides an internally filtered estimate of the q-axis current and the corresponding motor torque, which is used in this work as an actuator-side torque signal. This filtering reduces measurement noise at the expense of bandwidth, a trade-off that is acceptable for the intended quasi-static and low-frequency estimation.

Using a virtual-work formulation, the linear force applied to the actuation rod can be expressed as

4 $$\begin{aligned} F_x(\theta _1) = \frac{\eta _{\textrm{tot}}}{\rho _{\textrm{eff}}} \frac{\tau _m}{J_{x\theta }(\theta _1)}, \end{aligned}$$

where the scalar Jacobian $J_{x\theta }(\theta _1)=\textrm{d}x/\textrm{d}\theta _1$ captures the nonlinear kinematic mapping of the capstan–linkage mechanism and evaluates to

5 $$\begin{aligned} J_{x\theta }(\theta _1) = -\ell _1 \sin \theta _1 + \frac{ \ell _1 \cos \theta _1 \left( z_{\text {off}} - \ell _1 \sin \theta _1 \right) }{ \sqrt{ \ell _2^2 - \left( z_{\text {off}} - \ell _1 \sin \theta _1 \right) ^2 } }. \end{aligned}$$

Under field-oriented control, the electromagnetic motor torque is approximated by

6 $$\begin{aligned} \tau _m \approx k_t\, i_q, \end{aligned}$$

where $i_q$ denotes the filtered q-axis motor current and $k_t$ the motor torque constant. Substituting Eq. (6) into Eq. (4) yields the actuator-side force estimate

7 $$\begin{aligned} F_x(\theta _1, i_q) = \frac{\eta _{\textrm{tot}}\,k_t\, i_q}{\rho _{\textrm{eff}}\,J_{x\theta }(\theta _1)}. \end{aligned}$$

The efficiency term $\eta _{\textrm{tot}}$ serves as a lumped parameter capturing unmodeled losses between motor shaft and actuation rod, including friction and compliance in the capstan stage and linkage. In the current prototype this term has not yet been experimentally identified and is treated as an aggregated uncertainty parameter. It is not expected to be constant over the operating range, likely exhibiting position-, velocity-, and direction-dependent behavior due to the transverse forces the linkage geometry imposes on the linear guide. Systematic identification is planned by simultaneously recording motor torque and linear output force via the inline force sensor, allowing $\eta _{\textrm{tot}}$ to be mapped as a function of position and actuation direction. As a further consequence of the high-mechanical-advantage configuration near jaw closure, the mechanism approaches a near-self-locking condition in the static holding state: the back-driven force from the grasped tissue cannot easily reverse the linkage, allowing the motor to sustain grip force at significantly reduced torque demand. This behavior is intentional and beneficial for prolonged grasping, as it reduces motor effort and thermal load during sustained tissue holding. It should further be noted that as the linkage approaches this configuration, the Jacobian $J_{x\theta }$ becomes small, causing the force estimate of Eq. (7) to become increasingly sensitive to torque measurement noise and model uncertainty. This is a further motivation for the inline force sensor, which will provide a direct, more precise, and reliable output force measurement.

Position control of the linear drive relies on the motor encoder in combination with the known kinematic mapping. No dedicated linear position sensor is integrated in the current prototype. Within the assembled toolhead, force-related information is presently obtained from motor current and torque estimation.

The configurable nature of the actuation module enables the output force–stroke mapping to be tuned via kinematic parameter selection with respect to different grasping performance requirements, while the modular toolhead structure allows alternative drive units to be integrated and evaluated within the same framework.

#### Electronics and software framework

**Fig. 6:**
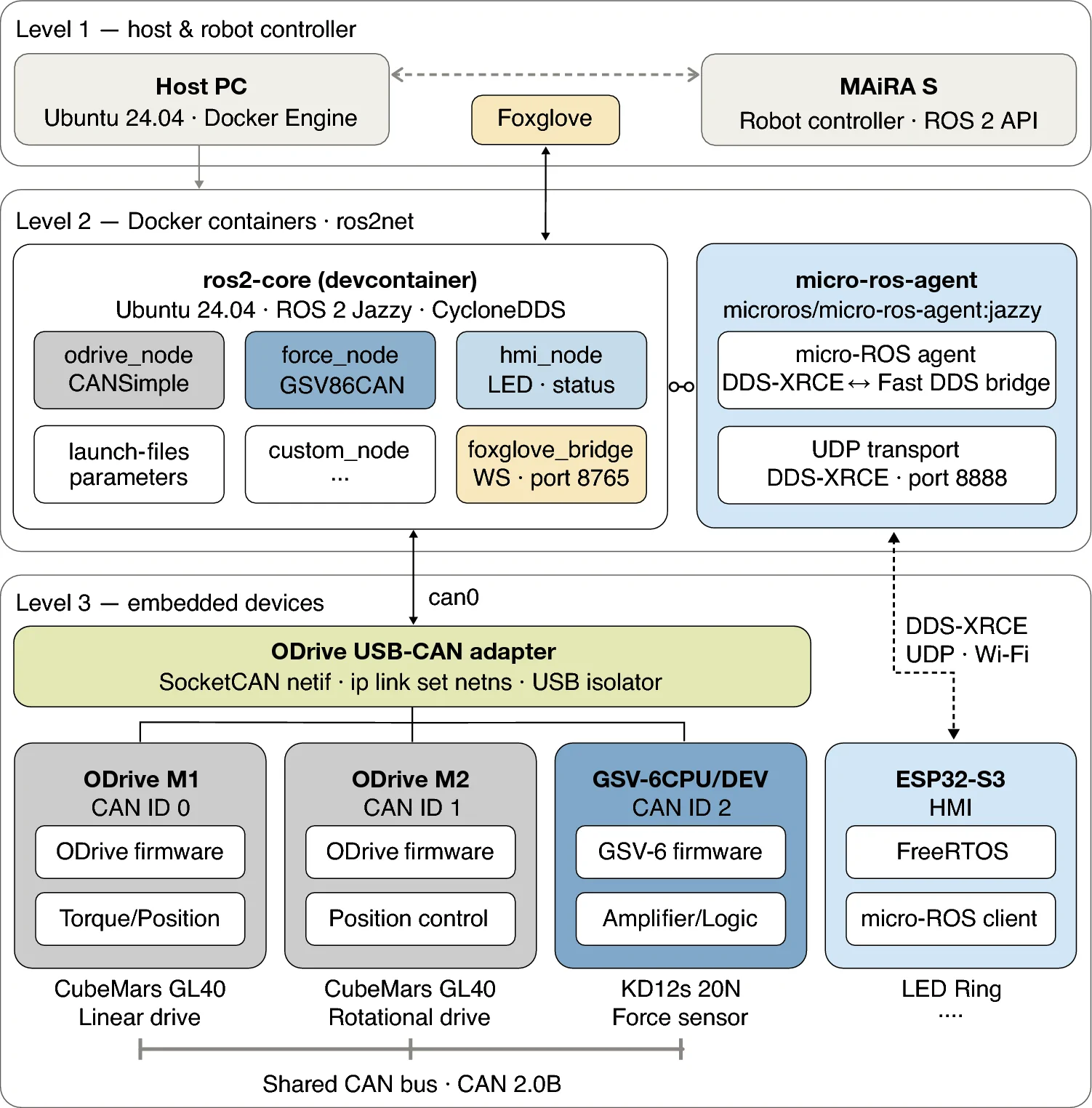
Software framework illustrating interfaces between embedded devices and the modular ROS control infrastructure

The electronic and software architecture of the developed end-effector was designed to support modular experimentation and integration into existing robotic systems, with a clear separation between low-level actuation and higher-level control visualized in Fig. 6. The toolhead is treated as a self-contained mechatronic subsystem that exposes standardized electrical and software interfaces.

Actuation is realized using gimbal-type brushless DC motors, specifically the CubeMars GL40 KV70 (Nanchang Kude Intelligent Technology Co., Ltd., Nanchang, China), driven by ODrive Micro (ODrive Robotics, Richmond, USA) motor controllers. Each controller implements cascaded current, velocity, and position control loops based on field-oriented control, using PI control for current and velocity regulation and proportional position control with feedforward terms. Rotor position feedback is provided via magnetic encoders: the linear actuation unit uses the on-board encoder of the motor controller, while the rotational actuation unit employs an external AS5048A encoder (ams-OSRAM AG, Premstaetten, Austria) connected via SPI. The same motor–controller configuration is used for both linear and rotational actuation, simplifying integration and accelerating software development. Low-level safety-related constraints are enforced locally at the motor controller level. Motor control and state feedback are handled through dedicated ROS 2 CAN interface nodes based on the ODrive CANSimple protocol [24]. The shared bus architecture allows additional CAN-based actuators, controllers, or sensing modules to be integrated by extending the existing network.

An architecture of two USB-C power delivery trigger boards separating the low-voltage and actuation electronics keeps the toolhead independent of robot-side power distribution, internal cable routing, and proprietary communication interfaces, allowing the system to be directly connected to a host computer for control and testing. The software framework is implemented using ROS 2 Jazzy and interfaces all hardware components through dedicated nodes. Motor controllers are accessed via CAN-based ROS 2 drivers, while higher-level logic such as synchronized motion commands, state machines, or interaction strategies is intentionally implemented outside the toolhead software. This structure allows task-specific control architectures to be developed, modified, and evaluated independently of the end-effector implementation.

For reproducible development and deployment, the main software components are containerized. System telemetry, actuator states, and sensor data are visualized using Foxglove (Foxglove Technologies Inc., San Francisco, USA), providing real-time insight during testing and evaluation. An ESP32-S3 microcontroller (Espressif Systems Co., Ltd., Shanghai, China) running micro-ROS manages toolhead user feedback by communicating wirelessly with a host-side Dockerized agent, publishing system status and driving a customizable LED ring mounted beneath the toolhead casing to indicate operational states and error conditions [25]. Additional sensors, input devices, or feedback modalities can be integrated through the microcontroller without affecting the actuation or communication architecture.

**Fig. 7:**
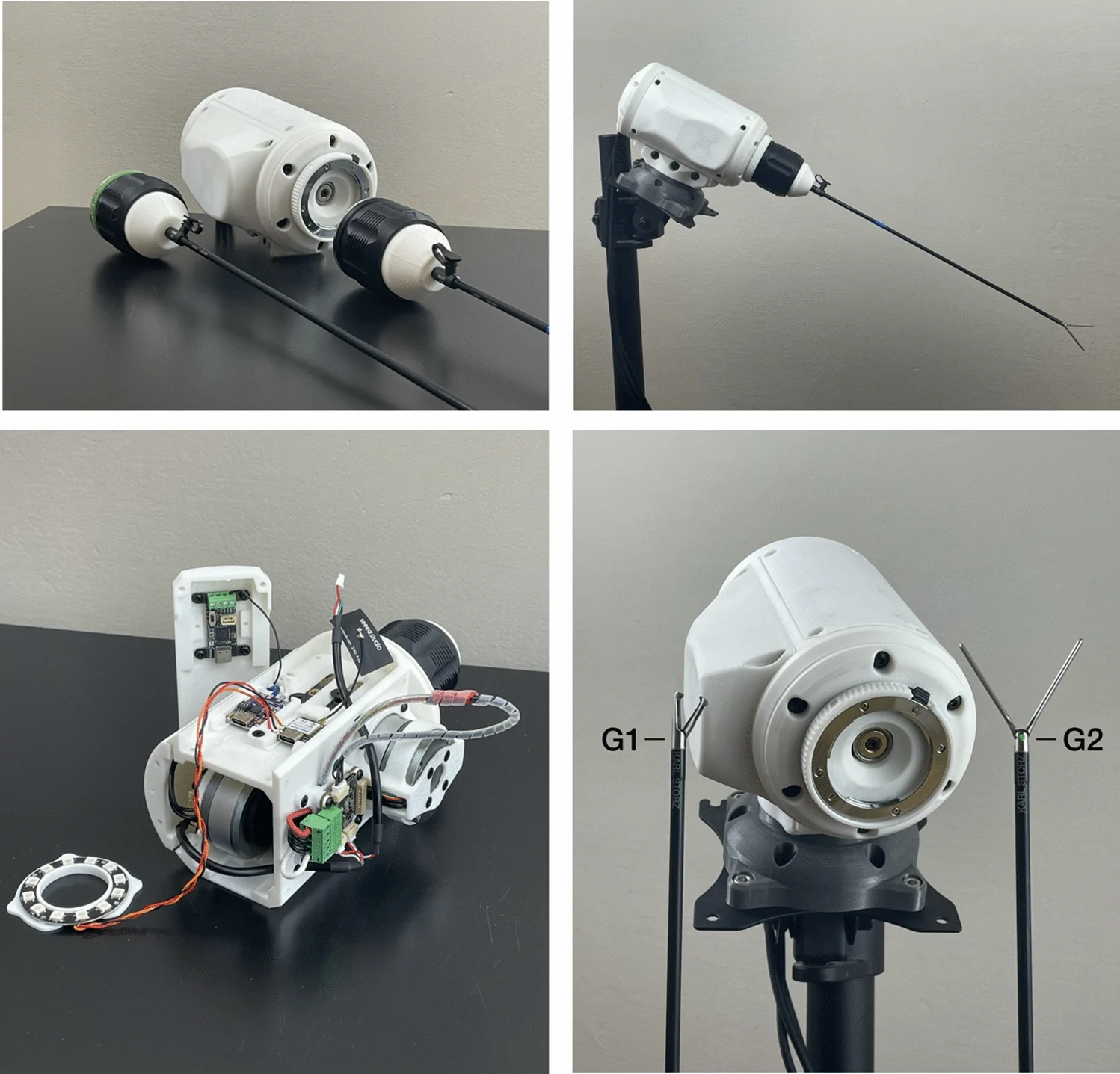
Final prototype of the robotic end-effector showing key structural features and integration highlights

### Verification

The verification of the toolhead is achieved through hierarchical functional testing of the actuation chain and the ROS 2 control framework, at both subsystem level and on the integrated assembly. Mechanical validation covers the actuation speeds of the linear and rotational drives, the endurance of the capstan linear drive under repeated loading, the function of the magnetic safety coupling, and preliminary grasping force characterization.

On the assembled toolhead, actuation speeds of the linear and rotational drives were obtained from ODrive encoder telemetry under a trapezoidal motion profile provided by the motor controller and exposed through ROS 2, timing the interval from trajectory command to target position over 20 repetitions per axis, deriving the mean execution time.

**Fig. 8:**
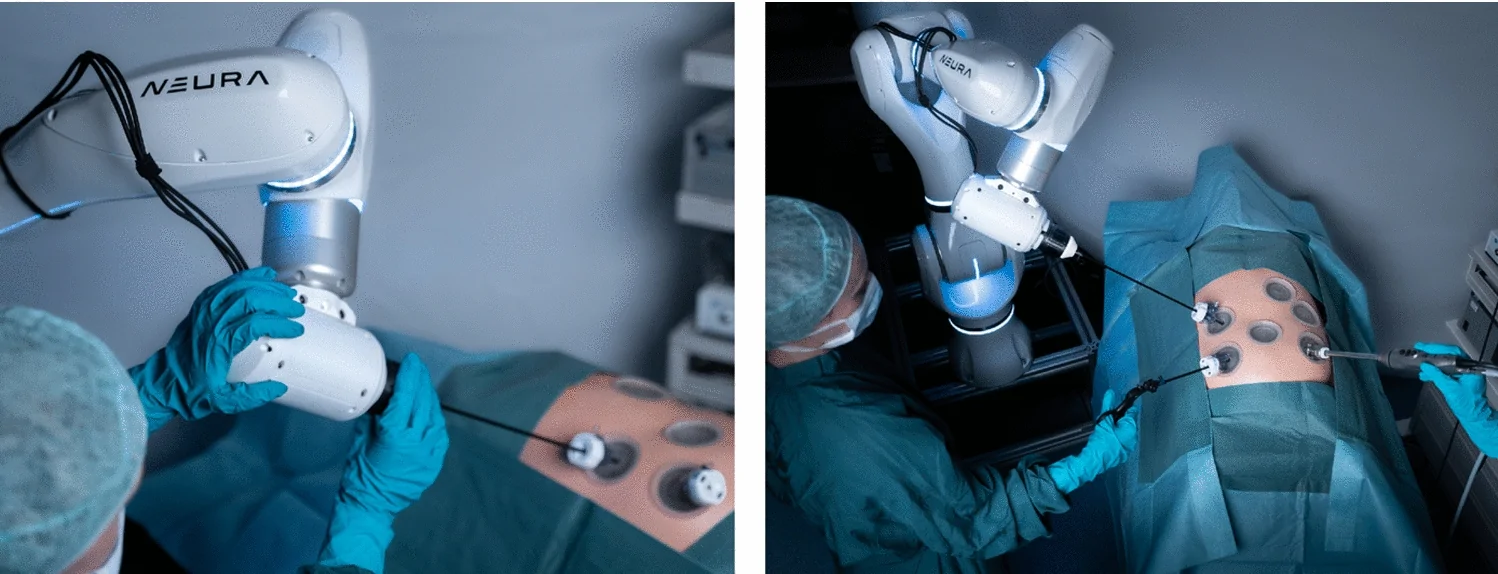
Experimental setup of the clinical test scenario for simulated cholecystectomy and sigmoid resection procedures, demonstrating the integration of the robotic toolhead into a standard laparoscopic operating environment

Endurance and preliminary grasping force measurements were performed on a test stand using the capstan linear drive as a functional unit extracted from the toolhead, together with an instrument interface. Endurance was assessed through 100 consecutive close–open cycles at approximately 50 N peak axial load, followed by visual inspection after disassembly of the instrument adapter and capstan stage. Grasping force measurements were performed with two Karl Storz CLICKLINE instruments: an atraumatic grasper with a single actuated jaw (G1) and a bowel grasper with a symmetric two-jaw mechanism (G2) shown in Fig. 7. The capstan linear drive actuated the instrument rod, and the resulting axial force was recorded by an inline KD12s load cell (ME-Meßsysteme, calibrated to 50 N), conditioned by a GSV-6CPU amplifier module on a GSV-6DEV carrier board providing the CAN transceiver, and transmitted over a CAN 2.0B bus. Grasping forces at the jaws were recorded using two strain-gauge load cells (5 kg range) read by an ADS1256 24-bit ADC with integrated amplification and an ESP32 microcontroller. The load cells were rigidly mounted to the test stand, and their connecting cables were held between the jaws at 14 mm from the center of rotation, corresponding to jaw opening angles of approximately 25$^{\circ }$ (G1) and 30$^{\circ }$ (G2). Ten close–open cycles were recorded per instrument, with the commanded rod force kept just below the inline sensor’s calibration limit. The mean forces for G1 and G2 are reported.

Software-side integration of the inline force sensor was validated during these measurements: the ODrive drives and the GSV-6 amplifier shared a single physical CAN 2.0B bus, with each device’s frames decoded by a dedicated ROS 2 odrive-node for the CANSimple protocol and force-node for the GSV protocol—demonstrating that a new sensing component could be integrated into the measurement chain without modification of the existing actuation stack.

The magnetic safety coupling was tested on the assembled toolhead by deliberately inducing overload conditions and verifying predictable disengagement and reproducible re-engagement. Interface compatibility is verified on the assembled toolhead via repeated instrument exchanges and multiple gripping and holding sequences on tissue-mimicking materials.

Furthermore, the integration into a clinical-like setup is tested. The robot is mounted on a mobile base platform for flexible positioning. The toolhead is mounted on the robot arm, and the laparoscopic instrument is introduced through a trocar into a synthetic tissue phantom placed on the table. A monitor displays real-time endoscopic video feedback. The arm was hand-guided via gravity compensation and the robot teach pendant for positioning, while instrument rotation and jaw actuation were commanded through the ROS 2 interface using Foxglove and a terminal. The reachability and orientation capabilities of the system were evaluated by manually guiding the instrument to multiple target locations on the phantom, emulating surgical tasks such as tissue grasping and presentation, and assessed through qualitative observations and informal feedback from a surgeon, clinical personnel, and robotics and computer-vision researchers.

## Results

This section summarizes the main outcomes of the experimental verification and the first system-level integration shown in Fig. 7. The primary goal of this work was to realize a compact and modular robotic toolhead that enables reliable actuation of conventional laparoscopic instruments and serves as a robust research platform for subsequent development and data-driven evaluation. The prototype operates as a self-contained mechatronic subsystem with repeatable instrument handling, stable multi-axis actuation, and a workflow-compatible integration concept. The resulting actuation unit has a length of 230 mm, a maximum diameter of 110 mm, and a mass of 1080 g.

### Functional verification

On the assembled toolhead, the linear drive achieved the required actuation travel to fully open and close the tested Karl Storz CLICKLINE graspers, while the rotation drive provided continuous and smooth instrument rotation. Coordinated two-axis operation was demonstrated through repeated opening/closing sequences under varying instrument orientations.

Gripping, holding, and controlled release were achievable, providing preliminary qualitative evidence of sufficient force transmission for representative laparoscopic assistance tasks. The quick-change interface enabled rapid instrument exchange and maintained stable coupling during repeated handling. The integrated magnetic safety coupling disengaged predictably under overload conditions and re-engaged reproducibly, supporting safe prototyping and protecting the instrument during unexpected load cases. In parallel, the electronics and ROS 2-based control framework operated robustly, enabling synchronized operation, system-state visualization, and the continuous recording of actuator-side signals. A first set of indicative performance metrics was obtained to support assessment of the system’s suitability for telemanipulated and autonomous operation. Under the applied trapezoidal profile, which was tuned for smooth actuation, the linear drive of the assembled toolhead completed a full grasper closing motion in under 0.5 s, and the rotation drive executed a 180$^{\circ }$ instrument rotation in approximately 1 s. These times correspond to the current operating set-point of the motion profile, chosen for reliable operation within the system’s requirements, and are not indicative of the drives’ maximum performance. On the dedicated test stand, the extracted capstan linear drive and instrument adapter sustained the 100-cycle endurance test without visible damage or rope slip. Across 10 grasping cycles per instrument, mean peak rod forces of 47.38 ± 0.40 N (G1) and 47.93 ± 0.34 N (G2) were recorded, with corresponding mean peak pinch forces of 6.54 ± 0.04 N (G1) and 4.59 ± 0.02 N (G2). A full characterization of force transmission, efficiency, and angle dependence is the subject of ongoing work.

### Phantom and clinical-like setup integration

The preliminary evaluation visualized in Fig. 8 provided valuable insights into the practical integration of the robotic toolhead within a surgical setting. Key observations highlighted the importance of compact robot positioning to avoid interference with the surgical team, the necessity for intuitive control interfaces like gravity compensation to support surgeon interaction, and the relevance of safety and feedback mechanisms for clinical acceptance. These integration results substantiate the feasibility of the developed toolhead as a modular research platform for future studies on controlled traction, motion strategies, and quantitative force/interaction characterization in realistic laparoscopic scenarios.

## Discussion

### Platform capabilities

The toolhead concept is not limited to the specific robotic platform and can be transferred to other remote controlled, robotic application scenarios. It provides a practical foundation for investigating autonomous and semi-autonomous assistance in laparoscopic surgery. By implementing grasping and axial instrument rotation directly at the end-effector level, the system decouples fine manipulation from the global motion of the robotic arm. Actuation, sensing, and interface components can be adapted independently, allowing targeted refinements without redesigning the entire system. This flexibility supports rapid iteration and parallel investigation of control and autonomy concepts, making the platform well suited for sustained research use rather than task-specific optimization. Additionally, the modular separation of the actuation unit and the instrument interface via a bayonet coupling allows for sterile draping of the actuators, while the adapter can be sterilized or designed as a single-use component. A key strength of the presented approach lies in its compatibility with standard laparoscopic instruments. Avoiding instrument modification preserves established sterilization processes, handling characteristics, and clinical familiarity, which simplifies experimental deployment and improves translational relevance. The use of conventional instruments also enables direct comparison between manual and robotic execution of identical tasks, providing a consistent basis for evaluating the performance of robotic assistance.

#### Limitations

Several limitations should be considered when interpreting the presented results. The experimental evaluation primarily relied on qualitative observations and functional verification. While this restricts quantitative performance comparison, it was sufficient to confirm feasibility, modularity, and simulated workspace compatibility, which were the primary goals of this development stage. Nevertheless, the architecture continuously visualizes and records actuator-side signals, and the inline force sensor has already been validated on a dedicated test stand and in software; its integration into the assembled toolhead and systematic force characterization remain future work. Evaluations were performed in phantom-based environments. Although this limits direct clinical inference, such settings are appropriate and necessary for early-stage investigation of robotic manipulation and autonomy under safety-critical constraints. From a hardware perspective, the prototype relies largely on additively manufactured polymer components, which enable rapid and cost-effective iteration but introduce compliance, wear, and tolerance effects. These material limitations constrain achievable stiffness and long-term durability and should be addressed in future iterations through higher-performance polymers or metallic components. The system currently supports single-degree-of-freedom grasping instruments. While sufficient for the targeted traction and presentation tasks, more complex manipulation would benefit from additional distal degrees of freedom (e.g. wristed instrument designs). The interface concept does not allow for such extensions, and further actuation and sensing integration would be necessary. Finally, the chosen architecture prioritizes adaptability and research flexibility, resulting in a larger form factor than highly integrated, task-specific designs.

## Conclusion

This work presented the design and evaluation of a modular robotic toolhead for actuating standard laparoscopic instruments. The system combines grasping actuation, axial instrument rotation, extensible electrical interfaces, and a robust software framework within a compact end-effector suitable for integration with robotic platforms. Functional testing confirmed reliable instrument actuation and predictable overload behavior, while integration in a laparoscopic phantom setup demonstrated compatibility with established workspace configurations. Future work will focus on integrating calibrated force sensing into the assembled toolhead, quantitative performance evaluation, and the systematic investigation of autonomy concepts such as adaptive grasping, controlled traction, and surgeon-initiated assistance, advancing the platform toward clinically relevant robotic support in minimally invasive surgery.

## References

[1] Patil MJ, Gharde P, Reddy K, Nayak K (2024) Comparative analysis of laparoscopic versus open procedures in specific general surgical interventions. Cureus 16(2):54433. 10.7759/cureus.54433 PMC10951803 38510915

[2] Klodmann J, Schlenk C, Hellings-Kuß A, Bahls T, Unterhinninghofen R, Albu-Schäffer A, Hirzinger G (2021) An introduction to robotically assisted surgical systems: current developments and focus areas of research. Curr Robot Rep 2(3):321–332. 10.1007/s43154-021-00064-3

[3] Sandoval J, Laribi MA, Faure JP, Brèque C, Richer JP, Zeghloul S (2021) Towards an autonomous robot-assistant for laparoscopy using exteroceptive sensors: feasibility study and implementation. IEEE Robot Autom Lett 6(4):6473–6480. 10.1109/LRA.2021.3094644

[4] Wah JNK (2025) The rise of robotics and ai-assisted surgery in modern healthcare. J Robot Surg 19(1):311. 10.1007/s11701-025-02485-0 PMC12181090 40540146

[5] Wee IJY, Kuo L-J, Ngu JC-Y (2020) A systematic review of the true benefit of robotic surgery: ergonomics. Int J Med Robot Comput Assist Surg 16(4):2113. 10.1002/rcs.2113 32304167

[6] Azizian M, Liu M, Khalaji I, DiMaio S (2018) The da Vinci surgical system. In: The Encyclopedia of medical robotics, vol 4. World Scientific, Singapore, pp 3–28. 10.1142/9789813232266_0001

[7] Zhang D, Xiao B, Huang B, Zhang L, Liu J, Yang G-Z (2019) A self-adaptive motion scaling framework for surgical robot remote control. IEEE Robot Autom Lett 4(2):359–366. 10.1109/LRA.2018.2890200

[8] Ngu JC, Tsang CB, Koh DC (2017) The da Vinci xi: a review of its capabilities, versatility, and potential role in robotic colorectal surgery. Robot Surg Res Rev 4:77–85. 10.2147/RSRR.S119317 PMC6193435 30697566

[9] Stephens TK, O’Neill JJ, Kong NJ, Mazzeo MV, Norfleet JE, Sweet RM, Kowalewski TM (2019) Conditions for reliable grip force and jaw angle estimation of da Vinci surgical tools 14(1):117–127. 10.1007/s11548-018-1866-8 30288699

[10] Schäfer MB, Friedrich GR, Pott PP (2022) Universal mechanical interface for surgical telemanipulation using conventional instruments. In: New trends in medical and service robotics. Springer, Cham, pp 107–114. 10.1007/978-3-030-76147-9_12

[11] Attanasio A, Scaglioni BD, Momi E, Fiorini P, Valdastri P (2021) Autonomy in surgical robotics. Robot Auton Syst 4:651–679. 10.1146/annurev-control-062420-090543. (Annual Review of Control)

[12] Younis R, Yamlahi A, Bodenstedt S, Scheikl P, Kisilenko A, Daum M, Schulze A, Wise PA, Nickel F, Mathis-Ullrich F, Maier-Hein L, Müller-Stich B, Speidel MS, Weitz J, Wagner M (2024) A surgical activity model of laparoscopic cholecystectomy for co-operation with collaborative robots. Surg Endosc 38(8):4316–4328. 10.1007/s00464-024-10958-w PMC11289174 38872018

[13] Stabenow S (2022) Development and design of an interface for laparoscopic instruments for hybrid surgery. Chair of sensor-based robotic systems and intelligent assistance systems. Technical University of Munich (TUM), Munich, Germany (Master’s thesis)

[14] Dobbelsteen JJ, Lee RA, Noorden M, Dankelman J (2012) Indirect measurement of pinch and pull forces at the shaft of laparoscopic graspers. Med Biol Eng Comput 50:215–221. 10.1007/s11517-012-0862-3 PMC3298650 22258638

[15] Khadem SM, Behzadipour S, Mirbagheri A, Farahmand F (2016) A modular force-controlled robotic instrument for minimally invasive surgery: efficacy for use in autonomous grasping against variable pull forces. Int J Med Robot Comput Assist Surg 12(4):620–633. 10.1002/rcs.1727 26804489

[16] Schäfer MB (2024) Extrakorporale messung von interaktionskräften zur darstellung mit einem seilgetriebenen haptischen eingabegerät für die roboterassistierte chirurgie. Ph.D. thesis, Universität Stuttgart. https://doi.org/10.18419/OPUS-14722

[17] Schäfer MB, Glöckner AM, Friedrich GR, Meiringer JG, Pott PP (2023) Measuring interaction forces in surgical telemanipulation using conventional instruments. Robotica 41(4):1335–1347. 10.1017/S0263574722001758

[18] Schäfer MB, Meiringer JG, Nawratil J, Worbs L, Giacoppo GA, Pott PP (2022) Estimating gripping forces during robot-assisted surgery based on motor current. Curr Dir Biomed Eng 8(1):105–108. 10.1515/cdbme-2022-0027

[19] Schäfer MB, Riepe S, Parenzan M, Bauer CJE, Hotz J, Meiringer JG, Weiland S, Pott PP (2024) Characterization of dynamic gripping force feedback for robot-assisted surgery. Curr Dir Biomed Eng 10(2):62–65. 10.1515/cdbme-2024-1068

[20] VDI/VDE-Gesellschaft Mess- und Automatisierungstechnik: VDI/VDE 2206: Entwicklung Mechatronischer und Cyber-physischer Systeme. Ersatz für Ausgabe 2004-06

[21] Macenski S, Foote T, Gerkey B, Lalancette C, Woodall W (2022) Robot operating system 2: design, architecture, and uses in the wild. Sci Robot 7(66):6074. 10.1126/scirobotics.abm6074 35544605

[22] Hwangbo J, Tsounis V, Kolvenbach H, Hutter M (2018) Cable-driven actuation for highly dynamic robotic systems. In: 2018 IEEE/RSJ international conference on intelligent robots and systems (IROS), pp 8543–8550. 10.1109/IROS.2018.8593569

[23] Kirchhoff J, Stryk O (2017) New insights in synthetic fiber rope elongation and its detection for ultra lightweight tendon driven series elastic robots. In: 2017 IEEE international conference on advanced intelligent mechatronics (AIM), pp 64–69. 10.1109/AIM.2017.8013996

[24] ODrive Robotics (2024) CAN Protocol. ODrive Robotics, Richmond, CA, USA. ODrive Robotics. https://docs.odriverobotics.com/v/latest/manual/can-protocol.html. Accessed 20 Dec 2025

[25] Belsare K, Rodriguez AC, Sánchez PG, Hierro J, Kołcon T, Lange R, Lütkebohle I, Malki A, Losa JM, Melendez F, Rodriguez MM, Nordmann A, Staschulat J, Mendel J (2023). In: Koubaa A (ed) Micro-ROS. Springer, Cham, pp 3–55. 10.1007/978-3-031-09062-2_2

